# Supplementary material for: Silicon-chip-based mid-infrared dual-comb spectroscopy
Source: Nat Commun. 2018 May 14;9:1869. doi: 10.1038/s41467-018-04350-1 (PMC5951801; doi:10.1038/s41467-018-04350-1)
Supplement: Supplementary file 1 — Supplementary Information [file 41467_2018_4350_MOESM1_ESM.pdf]

Manuscript: Silicon-chip-based mid-infrared dual-comb spectroscopy

Authors: Yu *et al.*

Supplemental Material

Supplementary Note 1: Device details

A thermal oxidation-based 'etchless' process was used to fabricate the devices (details in the Method section in [1]). The ring resonator has a 100-μm radius. Three microresonators and two chips are used in this paper.

Devices used for Fig. 3 and Fig. 4:
Chip 1: microresonator1 has a coupling gap of 500 nm between the bus waveguide and the ring
Chip 2: microresonator2 has a coupling gap of 500 nm between the bus waveguide and the ring

Devices used for Fig. 5 and Fig. 6:
Chip 1: microresonator1 has a coupling gap of 500 nm between the bus waveguide and the ring
Chip 2: microresonator3 has a coupling gap of 550 nm between the bus waveguide and the ring

Supplementary Note 2: Baseline filtering

The free-space interferometer (~ 0.5 and 0.8 meter in each of the arms) is sensitive to any mechanical vibrations and environmental change which adds baseline noise to a single spectrum at relatively low frequencies. Figure 5a in main text is the time trace after putting a numerical high-pass filter (> 5 MHz) to filter the baseline fluctuation. The original time trace is plotted below:

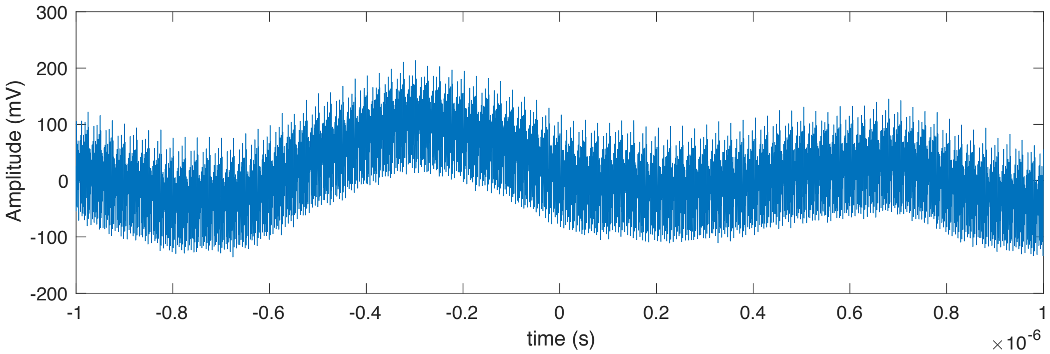

Supplemental Figure 1 **Measured dual-comb time trace over 2 μs**. The filtered data (> 5 MHz) is plotted in Fig.5a.

Supplementary Note 3: Optical resolution

The resolution of the dual-comb spectroscopy is determined by the comb line spacing of the modelocked frequency comb. The fit to M-FT data in Fig. SM1 gives an estimation of the comb line spacing to be 127.22 GHz ± 10 MHz.

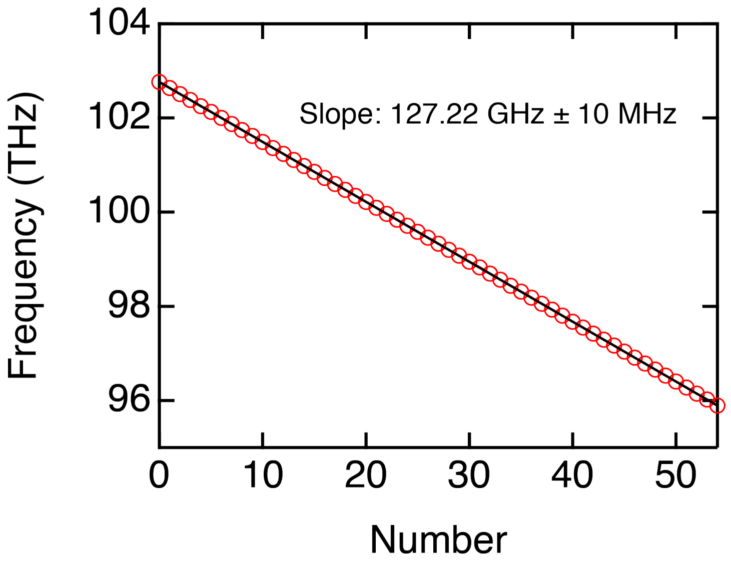

Supplementray Figure 2 **Characrterization of comb line spacing**. Measurement of comb line frequencies over 50 comb lines using M-FT.

Supplementary Note 4: Data analysis

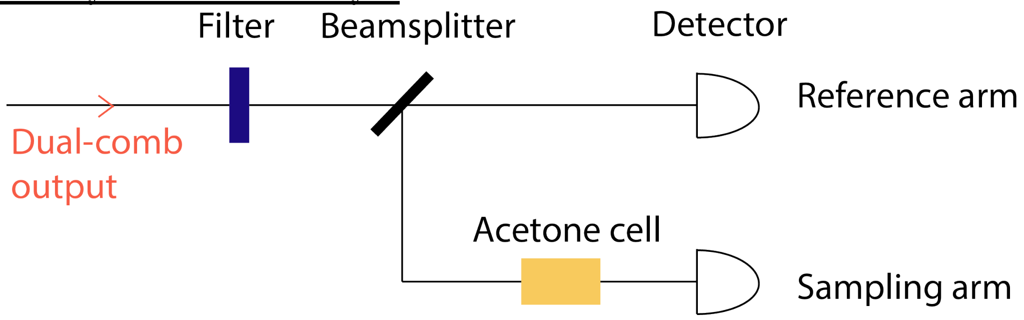

Supplementary Figure 3 **Setup for dual-comb measurement.** We split the dual-comb output and sent it to two mid-IR detectors (Vigo; PVI-4TE-8-1x1) to sequentially measure the cell transmission at the high and low wavelength sides with respective to the pump. The filter is placed before the beamsplitter. The high wavelength side is filtered with a bandpass filter centered at 3.25 µm with a bandwidth of 500 nm (Thorlabs FB3250-500). The low wavelength side is filtered with a bandpass filter centered at 2.72 µm (EO components IWBP2720-2980).

Detector calibration:
Two time traces (2µs each) are recorded with the two mid-IR detectors without inserting the cell.

1) High wavelengths side:

The FFT of the two time traces are shown below:

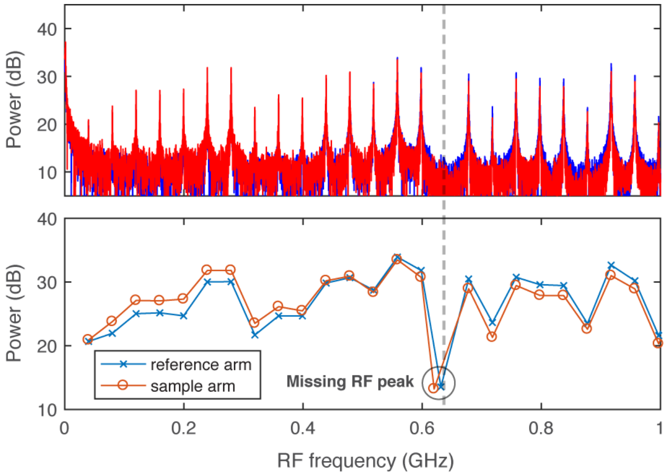

Supplementary Figure 4 **Detector calibration on the high wavelength side.** Top: the FFT spectra of time traces recorded by two detectors without the cell. Red: sampling arm. Blue: reference arm; Bottom: the extracted power of each RF peak. The circled point corresponding to 0.64 GHz is due to a missing RF peak in dual-comb spectra.

2) Low wavelengths side:

The FFT of the two time traces are shown below:

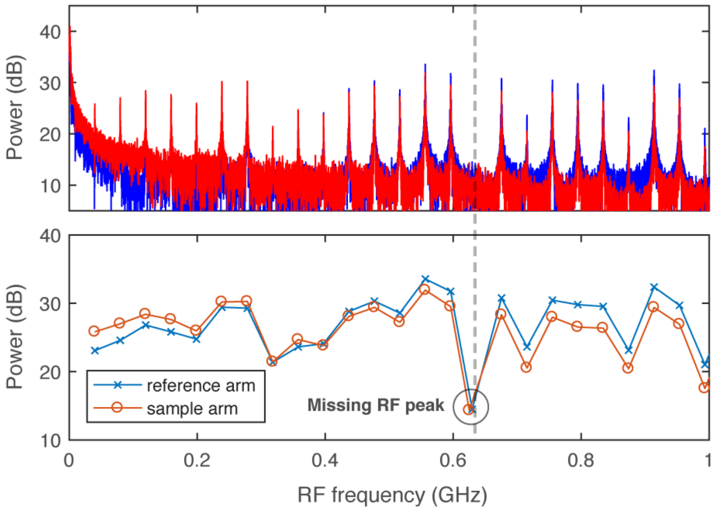

Supplementary Figure 5 **Detector calibration on the low wavelength side**. Top: the FFT spectra of time traces recorded by two detectors without the cell. Red: sampling arm. Blue: reference arm. Bottom: the extracted power of each RF peak. The circled point corresponding to 0.64 GHz is due to a missing RF peak in dual-comb spectra.

Absorption measurement:
We insert the 100 µm thick infrasil cell filled with acetone in the sampling arm. The infrasil cell has a 1.5 dB insertion loss across the measurement spectral window.

1) High wavelength side:

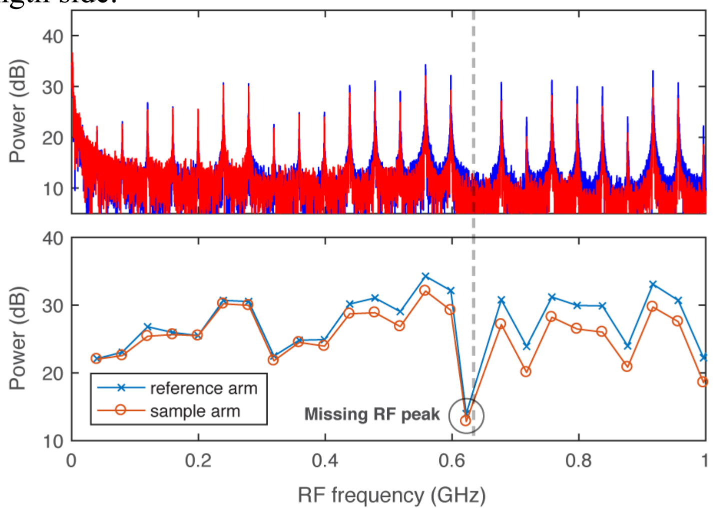

Supplementary Figure 6 **Absorption measurement of the acetone cell on the high wavelength side**. Top: the FFT spectra of time traces recorded by two detectors. Red: sampling arm. Blue: reference arm. Bottom: the extracted power of each RF peak. The circled point at 0.64 GHz is due to a missing RF peak in dual-comb spectra.

2) Low wavelength side:

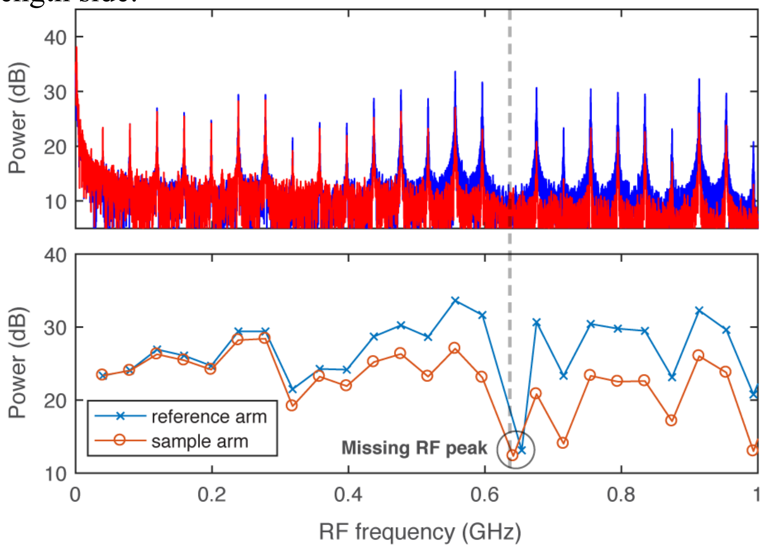

Supplementary Figure 7 **Absorption measurement of the acetone cell on the low wavelength side**. Top: the FFT spectra of time traces recorded by two detectors. Red: sampling arm. Blue: reference arm. Bottom: the extracted power of each RF peak. The circled point at 0.64 GHz is due to a missing RF peak in dual-comb spectra.

Combing the measurement and calibration on both sides (Supplementary Fig. 4-7), we plot the absorption measurement from the acetone cell:

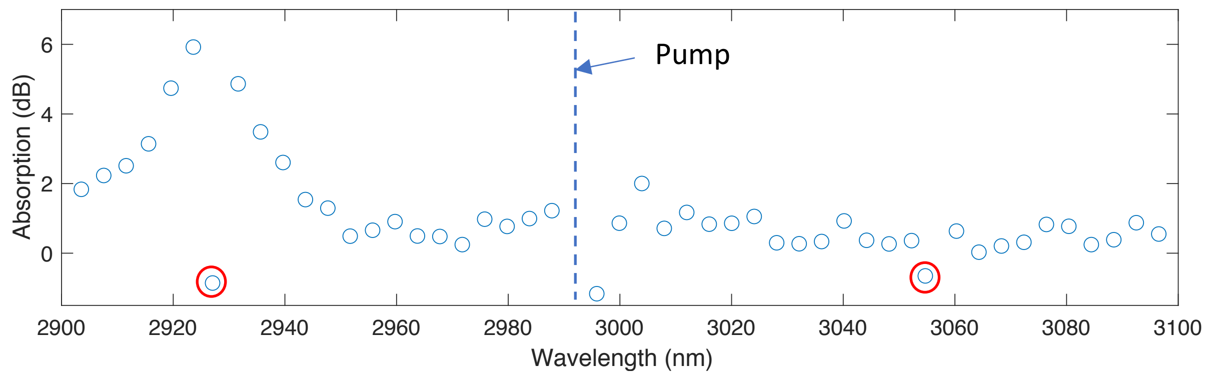

Supplementary Figure 8 **Absorption measurement of acetone**. Dashed line: the pump wavelength (2992 nm). The two circled points (2927 nm and 3055 nm) is due to missing RF peaks in dual-comb spectra as shown in previous plots.

Absorbance is absorption divided by 10, which corresponds to Fig. 6 in the main text. The absorption measurement near the pump shows a large deviation from the rest of the data points. We attribute this to a low SNR of dual-comb (<10dB) and the imperfect cut-off of the band pass filter.

<u>Supplementary Note 5:</u> Average SNR characterization

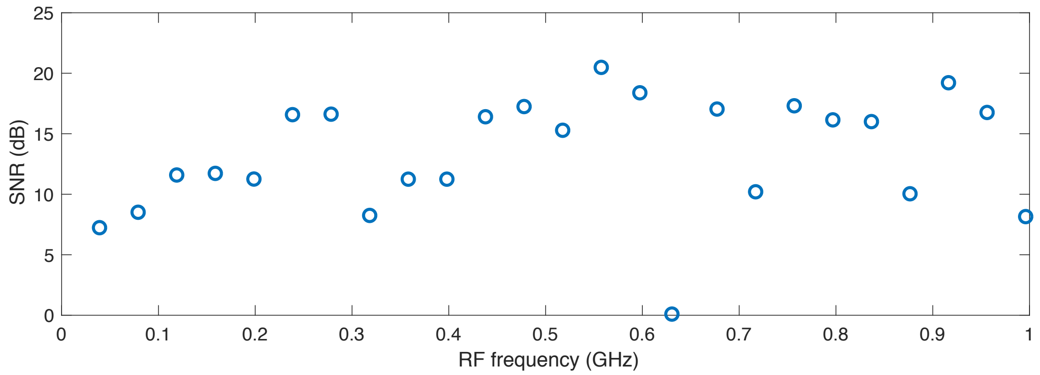

Supplementary Figure 9 **Signal to noise ratio of 25 RF beat notes based on Fig.5b**. The averaged SNR is 13.8 dB (≈ 40).

The SNR varies across the RF spectrum, as indicated in Fig.5b. We plot the SNR based on Fig. 5b and achieve an average SNR of 13.8 dB (excluding the 0.64 GHz point), which is responsible for the noise in our absorption measurement (4.1%) shown in Fig. 6b.

Supplementary References

[1] Griffith, A. G. *et al.* Silicon-chip mid-infrared frequency comb generation. *Nat. Commun*. **6**, 6299 (2015).
